# Sarcopenia and sarcopenic obesity among community-dwelling Peruvian adults: A cross-sectional study

**DOI:** 10.21203/rs.3.rs-3031470/v1

**Published:** 2023-06-13

**Authors:** Oscar Flores-Flores, Alejandro Zevallos-Morales, Suzanne L. Pollard, William Checkely, Trishul Siddharthan, John R. Hurst, Antonio Bernabé-Ortiz, Fernando M Runzer-Colmenares, Miles Witham, José F. Parodi

**Affiliations:** Universidad de San Martín de Porres; Prisma; Johns Hopkins University; Johns Hopkins University; University of Miami; University College London; Scientific University of the South; Scientific University of the South; Newcastle University; Universidad de San Martín de Porres

**Keywords:** Sarcopenia, muscle strength, muscle mass, sarcopenic obesity, older people

## Abstract

**Background:**

Sarcopenia and sarcopenic obesity (SO) have emerged as significant contributors to negative health outcomes in the past decade. However, there remains a lack of consensus on the criteria and cut-off thresholds for assessing sarcopenia and SO. Moreover, limited data are available on the prevalence of these conditions in Latin American countries. To address this evidence gap, we aimed to estimate the prevalence of probable sarcopenia, sarcopenia, and SO in a community-dwelling population of 1151 adults aged ≥ 55 years in Lima, Peru.

**Methods:**

Data collection for this cross-sectional study was conducted between 2018 and 2020 in two urban low-resource settings in Lima, Peru. Sarcopenia was defined as the presence of low muscle strength (LMS) and low muscle mass (LMM) according to European (EWGSOP2), US (FNIH) and Asian (AWGS) guidelines. We measured muscle strength by maximum handgrip strength; muscle mass using a whole-body single-frequency bioelectrical impedance analyzer, and physical performance using the Short Physical Performance Battery and 4-meter gait speed. SO was defined as a body mass index ≥ 30 kg/m^2^ and sarcopenia.

**Results:**

The study participants had a mean age of 66.2 years (SD 7.1), of which 621 (53.9%) were men, and 41.7% were classified as obese (BMI ≥ 30.0 kg/m^2^). The prevalence of probable sarcopenia was estimated to be 22.7% (95%CI: 20.3–25.1) using the EWGSOP2 criteria and 27.8% (95%CI:25.2–30.4) using the AWGS criteria. Sarcopenia prevalence, assessed using skeletal muscle index (SMI), was 5.7% (95%CI: 4.4–7.1) according to EWGSOP2 and 8.3% (95%CI: 6.7–9.9) using AWGS criteria. The prevalence of sarcopenia based on the FNIH criteria was 18.1% (95%CI: 15.8–20.3). The prevalence of SO, considering different sarcopenia definitions, ranged from 0.8% (95%CI: 0.3–1.3) to 5.0% (95%CI: 3.8–6.3).

**Conclusions:**

Our findings reveal substantial variation in the prevalence of sarcopenia and SO when using different guidelines, underscoring the necessity for context-specific cut-off values. Nevertheless, regardless of the chosen guideline, the prevalence of probable sarcopenia and sarcopenia among community-dwelling older adults in Peru remains noteworthy.

## Background

Sarcopenia is a complex syndrome defined as the pathological decrease of muscle quantity and quality (1, 2). Older adults are particularly at risk as physiological muscle loss through aging starts at approximately 40 years of age (3). Sarcopenia is associated with several adverse health outcomes including falls, disability, and death (4, 5). Similar negative impacts are associated with obesity, the prevalence of which has increased, particularly in low-middle income settings, posing social, economic and healthcare challenges (6). Obesity and sarcopenia are closely linked and might interact both pathologically and functionally. Obesity can independently lead to loss of muscle mass and function, due to metabolic derangements, sedentarism, and high co-occurence of non-communicable diseases (7). On the other hand, sarcopenia might promote fat accumulation due to reduced total energy expenditure. Infiltration of fat has been described in sarcopenia via inflammation and adipokine-related mechanisms (8). Thus, both might act synergistically to produce a vicious cycle of fat gain and muscle loss (7).

Unfortunately, assessment of both sarcopenia and sarcopenic obesity (SO) has methodological challenges. For sarcopenia, several working international groups (9, 10) have developed guidelines such as the European Working Group on Sarcopenia in Older People (EWGSOP2), the Foundation for the National Institutes of Health (FNIH), and the Asian Working Group for Sarcopenia (AWGS2). These guidelines focus on three main aspects: low muscle strength (LMS), low muscle mass (LMM), and low muscle performance (LMP) to classify older adults as sarcopenic and in some cases, severely sarcopenic. In each case (LMS, LMM and LMP), the guidelines use different parameters and cut-off points. For SO, ESPEN (European Society for Clinical Nutrition and Metabolism) and the European Association for the Study of Obesity (EASO), proposed that SO is defined as the co-existence of excess adiposity and LMS/function.

Latin America has one of the highest growth rates for adults aged ≥ 60 years (11), with a faster rise in obesity prevalence than the rest of the world (12). Unfortunately, there are limited estimates of the prevalence of sarcopenia and SO in this population. Studies that included application of the European (EWGSOP2) cut-offs for muscle strength demand careful consideration of different morphological and nutritional aspects that might influence muscle mass and strength (13–15), yet included calculation of muscle mass using formulas validated only in Caucasian populations (16), or used indicators of muscle mass that are no longer recommended e.g., calf circumference (17, 18).

Thus, our aim was to estimate the prevalence of probable sarcopenia (refering only to LMS, also called dynapenia), sarcopenia and SO in a representative community-based sample of adults ≥ 55 years old from Lima, Peru. Due to the methodologcal challenges in defining these conditions, we compared sarcopenia prevalence according to EWGSOP2, FNIH and AWGS2 criteria in our sample. Finally, to make useful and fair prevalence comparisons with similar settings, we compared the prevalence of probable sarcopenia and sarcopenia in selected Latin American countries applying the same criteria and definition of sarcopenia in our sample.

## Methods

### Study design and setting

This was a cross-sectional study nested in a large multi-national community-based project called the Global Excellence in Chronic Obstructive Pulmonary Disease Outcomes (GECo) study (19). At the Lima-Peru site, GECo enrolled an age and sex-stratified random community sample of 3,551 individuals aged 40 years and above, from two urban low resource settings of Lima. The inclusion and exclusion criteria for the GECo study is described elsewhere (20). During the study home-visit, participants performed physical tests (hand grip strength, short-physical performance battery), and socioeconomic and health surveys were administered. The study visit was performed at the participant’s home, except for a few cases (< 15) that were in a research office.

### Study sample and participants

**Figure 1:**
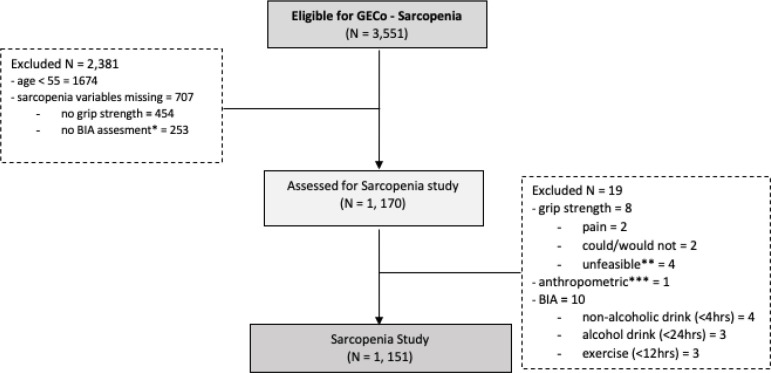
Flowchart of study participants * Reasons for No BIA assessment: (Unable = 25, Refused = 4, Architecture = 154, Missing = 70) ** Unfeasible events *** Weight less than 25 kg

For the present analysis, we selected a subset of participants who were ≥ 55 years old, performed handgrip strength testing and underwent bioelectrical impedance analysis (BIA) for body composition. We excluded from the analysis those who could not perform the handgrip test due to current wrist pain in the dominant hand or could not undergo BIA due to lack of adequate space at the participant’s home. Additionally, to increase the accuracy of BIA results, we excluded from the analysis those who had exercised in the last 12 hours and/or had consumed alcohol in the last 24 hours. Figure 1 shows a flow diagram of the enrolment of the participants.

### Variable measurements

#### Muscle strength

To measure muscle strength, participants performed three handgrip strength trials using a Jamar hydraulic dynamometer while sitting down [*Additional file 1- SOP dynamometry SOP*]. The dominant hand was determined with the question *“Which hand do you use to hold a fork to eat, or sign your name?”*. To define LMS the best trial had to be lower than the cut-off points from these definitions: EWGSOP2 (< 27 kg for men; <16 kg for women), FNIH (< 26 kg for men; <16 kg for women) and AWGS2 (< 28 kg for men; <18 kg for women). The best trial was used since it is less likely to be affected by the number of trials compared to the mean of the trials (21).

#### Muscle quantity

Appendicular skeletal muscle mass (ASMM,kg) was estimated using the whole-body single-frequency bioelectrical impedance analyzer BodyStat^®^500 (Bodystat LTD, Douglas, Isle of Man, UK) with the participant in supine position. The resistance (R) at 50 kHz was obtained and ASMM was calculated using the following formula for a non-Caucasian older population (22):

$$ASMM\;(kg)=-0.05376+\left(0.2394\;*\;\frac{H^{2}}{R}\right)+(2.708\;*\;sex)+(0.065\;*\;W)$$

where height (H) is measured in centimeters; BIA resistance (R) is measured in ohms (Ω), and weight (W) is measured in kg; for sex, men = 1 and women = 0. Additionally, we calculated Skeletal Muscle mass Index (SMI) as ASMM/height^2^.

To define low muscle mass (LMM), we used 1) the EWGSOP2 criteria for ASMM (ASMM < 20 kg & <15 kg), and SMI values (< 7.0 kg/m^2^ & <5.5 kg/m^2^), for men & women, respectively; 2) the FNIH criteria using reference for ASMM (ASMM < 19.75 kg & <15.02 kg), and ASM adjusted by the body mass index (BMI) values (ASM/BMI < 0.789 & <0.512), for men & women, respectively, and 3) the AWGS2 criteria using reference for SMI values (< 7.0 kg/m^2^ & <5.7 kg/m^2^), for men and women, respectively.

#### Physical performance

To evaluate physical performance, we measured the Short Physical Performance Battery (SPPB) score. The SPPB is based on three timed tasks: standing balance, 4-meter gait speed, and chair stand tests (23). The timed results of each subtest are scored according to predefined cut-points for obtaining a global score ranging from 0 (worst performance) to 12 points (best performance) (23). Gait speed was measured at usual pace at 4-meter length using the mean of two tests.

Low physical performance (LPP) was defined using the cut-off points for gait speed, SPPB, and chair time. For the EWGSOP2 definition an SPPB score ≤ 8 or a gait speed ≤ 8 was used. (5) In the AWGS2 definition, an SPPB score ≤ 9 or a 5-chair stand test ≥12 seconds was used. FNIH does not have a definition of LPP, since this guideline does not define a category of severe sarcopenia.

#### Probable Sarcopenia, Sarcopenia and Sarcopenic obesity

Probable sarcopenia was defined as LMS based on EWGSOP2 and AWGS2 guidelines.

Sarcopenia was defined as the presence of LMS and LMM according to the EWGSOP2, FNIH and AWGS2 guidelines. Severe sarcopenia was defined as the presence of sarcopenia and LMP according to the EWGSOP2 and AWGS2 guidelines.

Height was measured three times with a SECA 213^®^ stadiometer, and weight three times with SECA 803^®^ scale, clothed without shoes. BMI (score and categorized by WHO guidelines; BMI ≤ 24.9 as normal, BMI of 25 to 29.9 as overweight, BMI of 30 to 34.9 as class I obesity, and BMI ≥ 35 as class II obesity or more, all measured in kg/m2). Sarcopenic obesity was defined using a BMI equal or greater than 30 kg/m2 and sarcopenia as defined in EWGSOP2 or AWGS2. We additionally included age, sex, and level of education (number of years on education and classified).

Finally, we compared probable sarcopenia of our Peruvian sample with other Latin American countries, matching each study criteria: LMS cut-off, grip strength calculation, age and sex. For this, we conducted a convenient review of literature, searching databases (PubMed and Google Scholar) for articles published in English or Spanish, with search tems “sarcopenia”, “probable sarcopenia”, “dynapenia” performed in Latin American countries. Since EWGSOP2 or AWGS2 were developed in 2019, we mainly focused in studies performed since January 2019. In countries were no EWGSOP2/AWGS2 were used, we admitted EWGSOP1 guidelines using their respective cut-off. Additinally, we sought publications from the references lists of identified papers. Using information from each of the studies methods section, we obtained prevalences of probable sarcopenia by sex, and utilized the same parameters for calculating probable sarcopenia in our sample.

### Data analysis

We performed a descriptive analysis with means and standard deviation for continuous variables and frequencies and proportions for categorical variables. Prevalence estimates of probable sarcopenia, sarcopenia, and SO were calculated for all included definitions (EWGSOP2, FNIH and AWGS2) with their 95% confidence intervals (CI). While the GECO multisite study was weighted based on census information, we did not include weighted analysis for the present study. Missing data was handled by complete case analysis. Statistical analysis was performed using STATA 17 statistical software (StataCorp LP).

### Ethics

All participants provided written informed consent. Ethics permissions were obtained from the University College London Research Ethics Committee, Johns Hopkins School of Medicine, and A.B. PRISMA in Peru.

## Results

### Characteristics of the sample

Our sample included 1151 participants with a mean age of 66.2 years (SD 7.1), 621 (54.0%) were men, 44.1% were overweight (BMI of 25 to 29.9kg/m2) and 41.7% were obese (BMI ≥ 30.0kg/m2). General characteristics of the sample are presented in Table 1.

### Muscle parameters

In Table 2, we show the mean values of the sarcopenia parameters and proportion of individuals with LMS, LMM, and LMP. The proportion of individuals selected as having LMS using handgrip ranged between 20.2–31.3%. Regarding the proportion of people with LMM, using Skeletal Muscle Index (SMI), the proportion ranged between 16.2–20.6%, with difference of LMM between among women when using the EWGSOP2 (SMI: 17.4% in women) compared to AWGS2 (SMI: 27.0% in women). Using FNIH classification (ASM/BMI), the proportion of LMM increased substantially to 81.6% (79.2% in men, and 84.3% in women). Regarding the proportion of individuals with LMP, gait speed and SPPB score cut-offs from EWGSOP2 produce large variations (42.2% vs 8.1%, respectively). In contrast, using SPPB and Chair Time cut-offs according to AWGS2, the proportion of LMP ranged between 11–12.9%, with differences between men (6.8–8.1%) and women (15.9–18.6%).

### Probable sarcopenia, sarcopenia and SO prevalence

In Table 3, we show the prevalence of probable sarcopenia, sarcopenia and sarcopenic obesity. The prevalence of probable sarcopenia was 22.7% (95% CI: 20.3% – 25.1%) and 27.8% (95% CI: 25.2% – 30.4%) when using EWGSOP2 and AWGS2 definitions, respectively; without statistical differences between men and women. Regarding sarcopenia, prevalence varied between 5.7–18.1% among the study sample. Considering only EWGSOP2 and AWGS2 classifications with SMI, the prevalence was between 5.7% (95% CI: 4.4% – 7.1%) and 8.3% (95% CI: 6.7% – 9.9%). Furthermore, the prevalence of sarcopenia between men and women was statistically different using AWGS2 (6.8% vs 10.2% respectively, p = 0.036) but not in EWGSOP2 (6.4% vs 4.9%, p = 0.264).

Finally, a range from 0.8 to 5.0% of our sample had sarcopenic obesity. When using EWGSOP2 (ASM definition), a statistical difference between men and women was identified (2.9% vs 7.5%, p < 0.001). When using SMI in both AWGS2 and EWGSOP2, the prevalences were around 1%, without differences between men and women.

### Prevalence comparison in Latin America

Table 4 shows the comparison of probable sarcopenia point prevalence with different Latin American countries. We used the same criteria used in the selected papers, and used those criteria in our dataset. In Chilean studies, a custom cut-off hand grip estimated for their population (27 kg men and 15 kg women) was used which produced a lower prevalence compared to our sample. In Colombia, prevalence wa much higher than in the current study population (46.5% vs 34.2%) using the EWGSOP2 cut-off.

## Discussion

### Main findings

In our community-dweeling sample of adults 55 years and over in Lima Peru, at least one in five adults had probable sarcopenia, the prevalence of which varied due to different cut-offs across guidelines (22.7–27.8%). Furthemore, the prevalence of sarcopenia was lower when using SMI as the muscle mass parameter (5.7% in EWGSOP2 and 8.3% in AWGS2) compared to ASM (18.1% in FNIH, 16.3% in EWGSOP2). Finally, we found a low prevalence of sarcopenic obesity (0.8–5.0%) despite having a large proportion of obese (BMI > = 30) individuals in our sample (41.7%).

We have determined the prevalence of two important conditions, sarcopenia and sarcopenic obesity in an urban community setting. We had the challenge of not having a local or Latin American guideline for definitions, similar to European or Asian context. Thus, we found heterogeneity of prevalence estimates due to different cut-off points, and parameters suggested to define sarcopenia, with even less consensus about sarcopenic obesity. In response, to enable fairer comparisons, we elaborated Table 4 using the same cut-off points and age-groups in comparative studies. In spite of this, important differences remain. In Colombia, it was reported that almost half of the sample (46.5%) of healthy older adults had probable sarcopenia (15) compared to 34.2% in our low-resource community sample. Part of the difference might be because the Colombian study used the mean between grip trials in both hands, and not the recommended maximum trial of the dominant hand (21). Other Latin American studies, in Chile, used X-ray absorptiometry (DEXA) which can lead to a more accurate assessment of muscle mass than BIA, but this study used local cut-off points that came from a cross-sectional study that did not evaluate prediction of outcomes (24, 25). In a Brazilian study, EWGSOP2 and FNIH cut-off points were evaluated based on the lowest 20th (26). Additionally, the formula used to calculate muscle mass (ASM) was standardized only in an Australian population. In an attempt to consolidate several findings, a systematic review from 31 studies in Brazil found a sarcopenia prevalence of 17%, combining clinical and community samples, with great heterogeneity, several definitions, and including measurements using BIA and DEXA (27).

### Research Implications

Our study highlights the challenges that underpin measurement of sarcopenia and SO in a Latin American setting where there is no regional or local guideline and cut points. This is a call for research investment and collaboration to pool data from Latin America, with the need to develop longitudinal studies that allows determination of valid local cut-offs for hand grip and muscle mass criteria (with BIA or other methods such as ultrasound) associated with negative health outcomes, value our heterogeinity due to different levels of urbanicity and populations living at high altitude. The second call is for transparency. We included the manuals we used for the procedures of hand grip strength and muscle mass. However, that is not common practice, and creates high variability. For instance, if hand grip was measured standing up or sitting. Finally, although the design of the study did no allow us to recommend which guideline should be used for a Latin American population, there is no clear justification to prefer the European (EWGSOP2) over the Asian cut-offs (AWGS). In Peru, due to several factors including height, Asian countries cut-offs may be more appropriate (28).

### Strengths and Limitations of our study

This study has several strengths. Our sample was a census-representative sample of community-dwelling adults, which allowed a better approximation of the community prevalence of sarcopenia, although we acknowledge that some potential participants were excluded because they were unable to perform spirometry and these might be at higher risk of sarcopenia. A second strength is the effort to measure the parameters of sarcopenia with accurate methods, i.e., muscle strength measured in three attempts using maximum trial and muscle mass by whole-body bioimpedance analysis with valid equipment. BIA that uses two electrodes in the supine position is more accurate compared to those obtained standing or with only one electrode. Furthermore, we used a BIA formula validated in a similar population in Mexico and not the most common Caucasian formulas used in several Latin American papers. Neverthless, BIA results vary markedly between measurement tools and populations – a given conversion equation is accurate only for a particular combination of measurement tool and population and use of an equation developed in a Mexican population may not provide accurate results in our population. Further population and tool specific validation studies in our population would be needed to address this issue. Another limitation is that a considerable number of participants did not have sarcopenia measures, which might lead to some selection bias. Finally, we do not weight analysis to calculate prevalence estimates, which might lead to some innacuracies in the estimates, although they do not invalidate comparison across guidelines.

### Conclusion and perspectives

There is a considerable variation in the prevalence of probable sarcopenia, sarcopenia and SO among community-dwelling adults 55 years and older from urban settings in Lima, Peru, as assessed according to several guidelines and parameters. In spite of this variation, there is a substantial burden of probable sarcopenia and sarcopenia, which highlights the need to consider preventive measures and interventions. It is imperative to continue producing high-quality evidence regarding sarcopenia from regions such as Latin America, especially longitudinal studies that allow clinicians and researchers to define tailored cut-offs for sarcopenia parameters, which ultimately will promote a better understanding of sarcopenia and its prevention and management in these populations.

## Figures and Tables

**Table 1:** General characteristics of the study sample by sex.

Variables	Total (n = 1,151)	Men (n = 621)	Women (n = 530)
**Age years, mean ± SD**	66.2 ± 7.1	66.4 ± 7.4	65.9 ± 6.8
**Age category, n (%)**			
55.0–64.9	521 (45.3)	286 (46.1)	235 (44.3)
65.0–74.9	473 (41.1)	232 (37.4)	241 (45.5)
75 or more	157 (13.6)	103 (16.6)	54 (10.2)
**Height in cm, mean ± SD**	153.3 ± 8.5	159.1 ± 6.0	146.6 ± 5.6
**Weight in kg, mean ± SD**	69.4 ± 11.9	72.7 ± 11.1	65.5 ± 11.6
**BMI, kg/m2**	29.5 ± 4.5	28.7 ± 3.8	30.5 ± 5
**BMI, classified (kg/m2)**			
Normal	163 (14.2)	95 (15.3)	68 (12.8)
Overweight	508 (44.1)	317 (51.1)	191 (36.0)
Class I obesity (BMI 30 to < 35)	344 (29.9)	173 (27.9)	171 (32.3)
Class II obesity or more (BMI 35 or more)	136 (11.8)	36 (5.8)	100 (18.9)
**Education in years, mean ± SD**	7.7 ± 4.2	8.8 ± 3.8	6.4 ± 4.3
**Education, categonzed**			
No education or incomplete primary school	330 (28.7)	118 (19.0)	212 (40.0)
Primary school (Complete)	252 (21.9)	116 (18.7)	136 (25.7)
High school	460 (40.0)	315 (50.7)	145 (27.4)
University or other higher education	109 (9.5)	72 (11.6)	37 (7.0)

**Table 2:** Parameters of sarcopenia, and proportions of low muscle strength, low muscle mass and muscle performance according to different definitions.

Variables	Total (n = 1,151)	Men (n = 621)	Women (n = 530)
**Sarcopenia parameters, mean ± SD**			
Handgrip strength, kg	26.3 ± 9.4	32.1 ± 8.3	19.6 ± 5.3
Handgrip strength/BMI	0.9 ± 0.4	1.1 ± 0.3	0.7 ± 0.2
Appendicular skeletal muscle mass (ASMM)	17.2 ± 4.4	20.2 ± 3.2	13.6 ± 2.4
Skeletal muscle mass index (SMI)	7.2 ± 1.3	8.0 ± 1.1	6.3 ± 1
ASM/BMI	0.591 ± 0.2	0.711 ± 0.1	0.451 ± 0.1
SPPB score (N = 1068)	10.9 ± 1.4	11.2 ± 1.2	10.6 ± 1.6
Gait Speed, m/s (N = 1108)	0.8 ± 0.2	0.9 ± 0.2	0.8 ± 0.2
Chair time, sec (N = 1097)	8.8 ± 2.7	8.3 ± 2.5	9.5 ± 2.8
**Low muscle strength (LMS), n (%)**			
EWGSOP2: Max grip*	261 (22.7)	151 (24.3)	110 (20.8)
EWGSOP2: Chair*	37 (3.5)	13 (2.3)	24 (4.9)
FNIH: Max grip	232 (20.2)	122 (19.7)	110 (20.8)
FNIH: Grip/BMI	360 (31.3)	191 (30.8)	169 (31.9)
AWGS2: Max grip	320 (27.8)	159 (25.6)	161 (30.4)
**Low muscle mass (LMM), n (%)**			
EWGSOP2: ASM	719 (62.5)	310 (49.9)	409 (77.2)
EWGSOP2: SMI	186 (16.2)	94 (15.1)	92 (17.4)
FNIH: ASM/BMI	939 (81.6)	492 (79.2)	447 (84.3)
FNIH: ASM	693 (60.2)	283 (45.6)	410 (77.4)
AWGS2: SMI	237 (20.6)	94 (15.1)	143 (27.0)
**Low muscle performance (LMP), n (%)**			
**EWGSOP2**			
Gait speed (N = 1108)	468 (42.2)	209 (34.9)	259 (50.9)
SPPB (N = 1068)	86 (8.1)	34 (5.9)	52 (10.6)
**AWGS2**			
SPPB (N = 1068)	138 (12.9)	47 (8.1)	91 (18.6)
Chair time (N = 1068)	117 (11.0)	39 (6.8)	78 (15.9)

* According to EWGSOP2, having LMS (either with grip strength or Chair stand up) is considered as probable sarcopenia.

**Table 3.** Prevalence of sarcopenia and severe sarcopenia according to EWGSOP2, FNIH and AWGS2 classification

Variables	Total (n = 1,151)	95% CI	Men (n = 621)	95% CI	Women (n = 530)	95% CI
**Probable Sarcopenia** *						
**EWGSOP2**						
Max handgrip strength	261 (22.7)	(20.3% – 25.1 %)	151 (24.3)	(20.9% – 27.7%)	110 (20.8)	(17.3% – 24.2%)
**AWGS** **						
Max handgrip strength	320 (27.8)	(25.2% – 30.4%)	159 (25.6)	(22.2% – 29.0%)	161 (30.4)	(26.4% – 34.3%)

**Sarcopenia**						
**EWGSOP2**						
Max handgrip strength + ASM	188 (16.3)	(14.2% – 18.5%)	97 (1 5.6)	(12.8% – 18.5%)	91 (17.2)	(13.9% – 20.4%)
Max handgrip strength + SMI	66 (5.7)	(4.4% – 7.1%)	40 (6.4)	(4.5% – 8.4%)	26 (4.9)	(3.1% – 6.8%)
**FNIH**						
Max handgrip strength + ASM/BMI	208 (18.1)	(15.8% – 20.3%)	110 (17.7)	(14.7% – 20.7%)	98 (18.5)	(1 5.2% – 21.8%)
**AWGS**						
Max handgrip strength + SMI	96 (8.3)	(6.7% – 9.9%)	42 (6.8)	(4.8% – 8.7%)	54 (10.2)	(7.6% – 12.8%)

**Sarcopenic Obesity**						
**EWGSOP2**						
Sarcopenia (ASM) + BMI>=30	58 (5.0)	(3.8% – 6.3%)	18 (2.9)	(1.6% – 4.2%)	40 (7.5)	(5.3% – 9.8%)
Sarcopenia (SMI) + BMI>=30	9 (0.8)	(0.3% – 1.3%)	6 (1.0)	(0.2% – 1.7%)	3 (0.6)	(−0.1% – 1.2%)
**AWGS**						
Sarcopenia (SMI) + BMI>=30	14 (1.2)	(0.6% – 1.9%)	7 (1.1)	(0.3% – 2%)	7 (1.3)	(0.3% – 2.3%)

* No FNIH definition for probable nor severe sarcopenia

** This definition is based on the description of possible sarcopenia in the primary care or community setting of the AWGS guideline Values are n (%). ASM, appendicular skeletal muscle mass; BMI, body mass index; SPPB, short physical performance battery.

**Table 4:** Comparison of probable sarcopenia prevalence among community older adults belongin to Latin American countries

Reference	Country	Year	Characteristics		Prevalence from selected study n (%)	Population from selected study	Prevalence in our sample n (%)	Our Sample total

(29)	Brazil	2022	Age: 60 or more	Total	45 (34.4)	132	246 (27.3)	901

(30)	Brazil	2021	Age: 73 or more	Total	72 (13.6)	549	113 (46.9)	241

(26)	Brazil	2021	Age: 60 or more	Total	316 (24.5)	1290	246 (27.3)	901

(15)	Colombia	2020	Age: 60 or more	Male	1041 (42.8)	5237	169 (33.9)	499
			
			Grip: Mean of attempts	Female	1393 (57.2)		139 (34.6)	402
			
				Total	2434 (46.5)		308 (34.2)	901

(31)	Chile	2022	Age: 65 or more	Total	58 (55.2)	105	201 (31.9)	630
			Grip: 27 kg (Male) & 15 kg (Female)					

(32)	Chile	2022	Age: 60 or more Grip: 27 kg	Male	146 (19.3)	2311	143 (28.66)	499
			
				Female	298 (19.2)		95 (23.63)	402
			
				Total	444 (19.2)		238 (26.42)	901
			(Male) & 15 kg (Female)					

(33)	Chile	2017	Age: 60 or more	Male	21 (6.6)	1006	143 (28.66)	499
			
			Grip: 27 kg (Male) & 15 kg (Female)	Female	44 (6.4)		95 (23.63)	402
			
				Total	65 (6.5)		238 (26.42)	901

(34)	Mexico	2018	Age: 50–89	Male	160 (78.8)	724	207 (33.3)	621
			
			Grip: 30 kg (Male) & 20 kg (Female)	Female	116 (22.3)		254 (47.9)	530
			
				Total	276 (38.1)		461 (40.1)	1151
			
			Age: 50–89	Male	27 (13.3)		207 (33.3)	621
			
			Grip: 29.1 kg (Male) & 18.4 kg (Female)	Female	66 (12.7)		229 (43.2)	530
			
				Total	93 (12.8)		436 (37.8)	1151

Unless specified, grip strength was measuread as best attempt and EWGSOP2 threshold values were used (< 27 kg for men; <16 kg for women)

## Data Availability

The dataset used in the current study is available from the corresponding author on reasonable request
